# Improvement of tissue preparation for laser capture microdissection: application for cell type-specific miRNA expression profiling in colorectal tumors

**DOI:** 10.1186/1471-2164-11-163

**Published:** 2010-03-10

**Authors:** Shuyang Wang, Lei Wang, Tengfang Zhu, Xue Gao, Jian Li, Ying Wu, Hongguang Zhu

**Affiliations:** 1 Department of Pathology, Shanghai Medical College, Fudan University, 138 Yi Xue Yuan Road, Shanghai 200032, PR China; 2 Department of Healthcare, Philips Research Asia - Shanghai, No 10, Lane 888, Tian Lin Road, Shanghai 200233, PR China; 3 Division of Surgical Pathology, Huashan Hospital, Fudan University, 12 middle-Wulumuqi Road, Shanghai 200040, PR China; 4 Research Center for Pathology, Institute of Biomedical Sciences, Fudan University, 138 Yi Xue Yuan Road, Shanghai 200032, PR China

## Abstract

**Background:**

Laser capture microdissection (LCM) has successfully isolated pure cell populations from tissue sections and the combination of LCM with standard genomic and proteomic methods has revolutionized molecular analysis of complex tissue. However, the quantity and quality of material recovered after LCM is often still limited for analysis by using whole genomic and proteomic approaches. To procure high quality and quantity of RNA after LCM, we optimized the procedures on tissue preparations and applied the approach for cell type-specific miRNA expression profiling in colorectal tumors.

**Results:**

We found that the ethanol fixation of tissue sections for 2 hours had the maximum improvement of RNA quality (1.8 fold, p = 0.0014) and quantity (1.5 fold, p = 0.066). Overall, the quality (RNA integrity number, RIN) for the microdissected colorectal tissues was 5.2 ± 1.5 (average ± SD) for normal (n = 43), 5.7 ± 1.1 for adenomas (n = 14) and 7.2 ± 1.2 for carcinomas (n = 44). We then compared miRNA expression profiles of 18 colorectal tissues (6 normal, 6 adenomas and 6 carcinomas) between LCM selected epithelial cells versus stromal cells using Agilent miRNA microarrays. We identified 51 differentially expressed miRNAs (p <= 0.001) between these two cell types. We found that the miRNAs in the epithelial cells could differentiate adenomas from normal and carcinomas. However, the miRNAs in the stromal and mixed cells could not separate adenomas from normal tissues. Finally, we applied quantitative RT-PCR to cross-verify the expression patterns of 7 different miRNAs using 8 LCM-selected epithelial cells and found the excellent correlation of the fold changes between the two platforms (R = 0.996).

**Conclusions:**

Our study demonstrates the feasibility and potential power of discovering cell type-specific miRNA biomarkers in complex tissue using combination of LCM with genome-wide miRNA analysis.

## Background

Molecular profiling of clinical tissue specimens is frequently complicated by their cellular heterogeneity. Laser capture microdissection (LCM) has successfully been used to tackle this problem by isolating pure cell populations from tissue sections [1-3] and the combination of LCM with standard genomic and proteomic methods has revolutionized molecular analysis of complex tissue. It has allowed for the discrimination of genomic changes, differential expressions and subsequent signaling effects for a variety of proteins in diagnostic tissues [4-10]. Despite these advances, the quantity and quality of material recovered after LCM is often still limited for analysis by using whole genomic and proteomic approaches [2,11].

MicroRNAs (miRNAs) play important regulatory roles in various cellular pathways including development, cell proliferation, differentiation and apoptosis [12-14]. Demonstrated abnormal expression patterns of miRNAs in human disease tissues highlight their potential use as diagnostic and prognostic biomarkers, especially in the case of cancer [15-20]. In fact, miRNAs have already been demonstrated to function as both tumor suppressors and oncogenes [21,22]. Furthermore, miRNAs have advantages over mRNAs as cancer biomarkers, since they are very stable *in vitro *[15] and long-lived *in vivo *[23]. So far, the large majority of published miRNA expression studies utilized whole tumor tissues without separating the truly transformed cancerous cells from those other cell types commonly present within a tumor (e.g. immune, stroma cells and new vasculature, etc). Analysis of such complex tissues could conceal the specific signature of the particular cell type of interest. A potentially powerful method to develop diagnostic tests would be to correlate cell type-specific miRNA profiles with pathologic and clinical outcomes.

Combination of LCM and whole genome analysis is an ideal method for cell type-specific expression profiling in complex tissue, however, such a combination has not been widely applied to discover miRNA biomarkers in solid tumors. To explore the possibility of using LCM for genome-wide miRNA analysis, we optimized the procedures on tissue preparation and then compared the miRNA expression profiles of 18 colorectal tissues in LCM selected epithelial cells and stromal cells using Agilent miRNA microarrays. We then applied quantitative RT-PCR to cross-verify the expression patterns of 7 different miRNAs using 8 LCM-selected epithelial cells. In this study, we demonstrate a significant improvement in RNA quality and quantity by prolonged ethanol fixation of tissue sections. We further present 51 significantly differentially expressed miRNAs between the epithelial and stromal cells from colorectal tissues. We then show that the miRNAs in the epithelial cells could differentiate adenomas from normal and carcinomas, however, the miRNAs in the stromal and mixed cells could not separate adenomas from normal tissues. We finally illustrate the correlation of the fold changes between the microarray and quantitative RT-PCR. To our knowledge, this work is the first demonstration of the feasibility and potential power of using a combination of LCM with genome-wide miRNA analysis on discovering cell type-specific miRNA biomarkers in complex tissue.

## Results

### Effect of ethanol fixation on RNA quality and quantity

**Figure 1:**
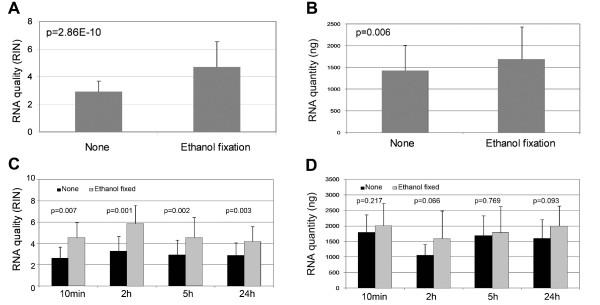
**Effect of ethanol fixation on RNA quality and quantity**. A) RNA quality (RIN scores) of the tissue sections in the presence (n = 24) and absence (n = 24) of ethanol fixation; B) RNA quantity (ng) of the tissue sections in the presence (n = 24) and absence (n = 24) of ethanol fixation; C) RIN scores of the tissue sections over four time points in the presence (n = 6 per time point) and absence (n = 6 per time point) of ethanol fixation and D) RNA quantity (ng) of the tissue sections over four time points in the presence (n = 6 per time point) and absence (n = 6 per time point) of ethanol fixation. Error bars indicate the corresponding SD. The large errors of the experiments were due to the fact that each tested group consisted of three different tissue types (normal, adenoma and carcinoma) which had the different RNA quality and quantity.

To assess the effect of ethanol fixation on RNA quality and quantity, we immediately fixed fresh tissue sections with 100% ethanol for 10 minutes, and then stored the slides at -80°C for 2, 5 and 24 hours. The experimental conditions and their corresponding RIN scores are shown in Table 1. RNA quality and quantity of these sections in presence and absence of ethanol fixation are displayed in Figure 1. Overall, the ethanol fixation significantly improved RNA quality (1.6 fold with p = 2.86E-10, Figure 1A) and quantity (1.2 fold with p = 0.006, Figure 1B). The maximum improvement of the quality (1.81 fold, p = 0.0014) and quantity (1.52 fold, p = 0.066) were observed in storing the sections with 100% ethanol at -80°C for 2 hours (Figures 1C and 1D).

**Table 1:** Experimental conditions for tissue preparations

Experiment	Section	Ethanol fixation	Storage at -80°C	Inhibitor*	Staining	LCM	RIN & SD
**Ethanol fixation (n = 48)**							

A-control	6	no	no	no	no	no	2.7 ± 0.5

A	6	10 min	no	no	no	no	4.8 ± 1.6

B-control	6	no	2 h	no	no	no	3.4 ± 0.8

B	6	10 min	2 h	no	no	no	6.1 ± 1.9

C-control	6	no	5 h	no	no	no	3.1 ± 1.0

C	6	10 min	5 h	no	no	no	4.9 ± 2.1

D-control	6	no	24 h	no	no	no	2.9 ± 0.7

D	6	10 min	24 h	no	no	no	4.4 ± 1.6

**Rnase inhibitor (n = 12)**							

E-control	6	no	no	no	no	no	3.4 ± 1.9

E	6	no	no	5 min	no	no	2.8 ± 0.9

**LCM (n = 22)**							

F-control	11	10 min	2 h	no	1 min	no	7.6 ± 0.8

F	11	10 min	2 h	no	1 min	yes	5.8 ± 1.4

*Add RNase inhibitor to hemotoxylin solution.

### Effect of RNase inhibitor on RNA quality and quantity

Besides the fixation, we evaluated the effect of RNase inhibitor treatment on the tissue preparation. RNA quality and quantity of the tissue sections in presence and absence of an RNase inhibitor are shown in Additional file 1. The presence of the RNase inhibitor reduced both RNA quality and quantity of one sample (S6), whilst slightly improved the RNA quantity in two samples (S1 and S5). Essentially, there was no considerable improvement in both quality and quantity of RNA recovered from the tissue sections with the inhibitor treatment.

### Effect of LCM on RNA quality

**Figure 2:**
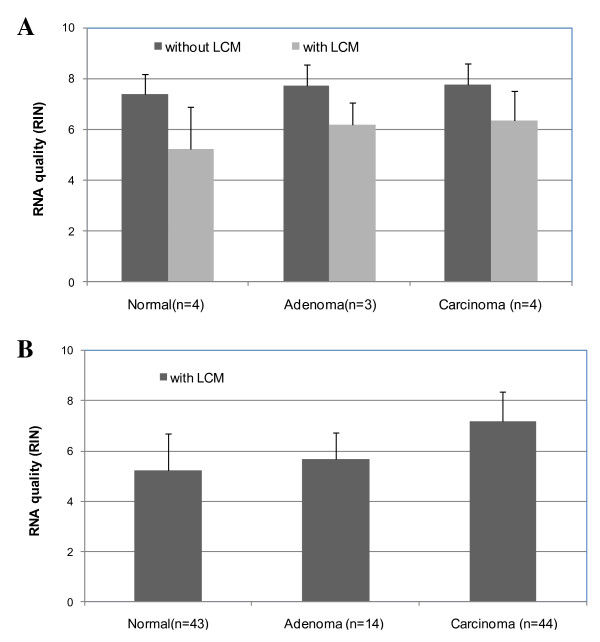
**Effect of LCM on RNA quality**. A) RNA quality (RIN scores) of the hematoxylin-stained sections with (n = 11) and without (n = 11) LCM and B) RNA quality (RIN scores) of the LCM selected epithelial cells derived from 43 normal, 14 adenoma and 44 carcinoma tissues. Error bars indicate the corresponding SD.

Using the improved protocol for tissue preparation, we first determined RNA quality in the hematoxylin-stained sections with and without LCM. All the samples were subjected to the same fixation and staining processes but the only difference was the use of microdissection. The RIN score was 7.6 ± 0.8 (average ± SD) for the sections without LCM and 5.8 ± 1.4 (average ± SD) for the sections with LCM (Figure 2A). Compared to the sections without LCM, the RIN score was decreased by 30% during the microdissection (p = 0.004). We then examined the consistency of RNA quality for the LCM selected epithelial cells derived from 101 colorectal tissues (43 normal, 14 adenomas and 44 carcinomas). On average, the RIN score was 5.2 ± 1.5 (average ± SD) for normal, 5.7 ± 1.1 (average ± SD) for adenoma and 7.2 ± 1.2 (average ± SD) for carcinoma tissues (Figure 2B).

### Reliability of LCM and miRNA analysis

**Figure 3:**
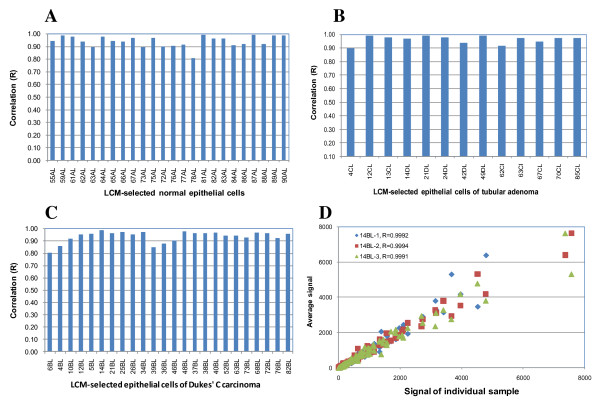
**Reliability of LCM and miRNA analysis**. A) correlation amongst individual samples of epithelial cells derived from 24 normal colorectal tissues; B) correlation amongst individual samples of epithelial cells derived from 13 colorectal tubular adenomas; C) correlation amongst individual samples of epithelial cells derived from 24 colorectal Dukes' C carcinomas and D) correlation amongst triplicate LCM experiments.

Replicate experiments were performed to determine the reliability of combining LCM with genome-wide miRNA analysis. The epithelial cells were microdissected on 61 individual colorectal tissues including 24 normal, 13 tubular adenomas and 24 Dukes' C carcinomas. We performed array hybridizations on these epithelial cells and determined the correlation amongst individual samples derived from the same tissue type (Figures 3A, 3B and 3C). The average correlation (R) of epithelial cells isolated from normal tissues, tubular adenomas and Dukes' C carcinomas was 0.942, 0.963 and 0.937, respectively. To determine the variability of the LCM protocol, we performed triplicate LCM experiments on the same tumor tissue and hybridized the LCM-selected epithelial cells on three individual microarrays. As shown in Figure 3D, the correlation (R) amongst the triplicate experiments was 0.999.

### Cell type-specific miRNA expression profiles

**Figure 4:**
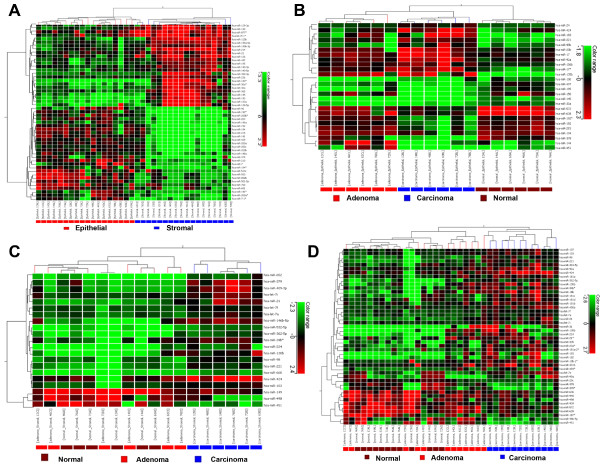
**Cell type-specific miRNA expression profiles**. A) hierarchical clustering of 51 miRNA expression profiles in LCM selected epithelial and stromal cells from 18 colorectal tissues (n = 6 normal, n = 6 adenomas and n = 6 carcinomas); B) hierarchical clustering of 26 miRNA expression profiles in LCM selected epithelial cells from the colorectal tissues; C) hierarchical clustering of 21 miRNA expression profiles in LCM selected stromal cells from the colorectal tissues and D) hierarchical clustering of 46 miRNA expression profiles in the mixed cell types (epithelial and stromal cells) from the colorectal tissues. The mean signal from biological replicate samples was used for the clustering. Colored bars indicate the range of normalized log2-based signals.

Using Agilent miRNA microarrays containing 723 human miRNA probe sets, we profiled the miRNA expression of 18 colorectal tissues in LCM selected epithelial and stromal cells. Significance analysis resulted in the identification of 51 miRNAs as differentially expressed between the epithelial and stromal cell types (Table 2). Figure 4A illustrates an unsupervised hierarchical clustering of these differentially expressed miRNAs and shows that the clustering placed 18/18 epithelial cells in one group and 18/18 stromal cells in another group. Expression levels of 723 human miRNAs in the epithelial and stromal cells of colorectal tissues are given in Additional file 2.

**Table 2:** Differentially expressed miRNAs in epithelial and stromal cells of colorectal tissues

Name	Geomean		Fold change	Unpaired t-test	Cell type
	Epithelial	Stromal	Epithelial/stromal	p-value	
hsa-miR-143	64	512	0.1	1.30E-06	Stromal

hsa-miR-145	512	4096	0.1	5.60E-06	Stromal

hsa-miR-133a	2	16	0.1	2.40E-07	Stromal

hsa-miR-139-5p	2	8	0.3	2.20E-05	Stromal

hsa-miR-125b	256	1024	0.3	2.60E-07	Stromal

hsa-miR-149	8	32	0.3	8.30E-09	Stromal

hsa-let-7f-1*	1	8	0.1	6.80E-06	Stromal

hsa-miR-143*	1	8	0.1	1.80E-06	Stromal

hsa-miR-30a	32	128	0.3	5.30E-07	Stromal

hsa-miR-214	64	256	0.3	2.80E-06	Stromal

hsa-miR-199a-5p	128	512	0.3	6.20E-07	Stromal

hsa-miR-195	128	512	0.3	3.10E-06	Stromal

hsa-miR-365	128	512	0.3	3.60E-07	Stromal

hsa-miR-136	2	8	0.3	1.10E-05	Stromal

hsa-miR-129-3p	2	8	0.3	6.60E-05	Stromal

hsa-miR-30a*	1	4	0.3	4.00E-05	Stromal

hsa-miR-497	64	256	0.3	2.30E-05	Stromal

hsa-miR-140-5p	32	128	0.3	3.70E-07	Stromal

hsa-miR-877*	8	16	0.5	2.10E-05	Stromal

hsa-miR-199b-3p	512	1024	0.5	7.70E-07	Stromal

hsa-miR-22	512	1024	0.5	9.40E-06	Stromal

hsa-miR-490-3p	1	2	0.5	8.90E-05	Stromal

hsa-miR-23b	1024	2048	0.5	4.10E-06	Stromal

hsa-miR-140-3p	64	128	0.5	4.50E-05	Stromal

hsa-miR-141*	2	1	2	6.90E-05	Epithelial

hsa-miR-7-1*	4	2	2	6.20E-05	Epithelial

hsa-miR-194*	4	1	4	2.90E-06	Epithelial

hsa-miR-760	8	2	4	7.20E-05	Epithelial

hsa-miR-513c	4	1	4	3.80E-05	Epithelial

hsa-miR-200a*	8	2	4	1.70E-06	Epithelial

hsa-miR-148a	512	128	4	1.30E-06	Epithelial

hsa-miR-501-5p	8	2	4	4.10E-07	Epithelial

hsa-miR-601	16	2	8	5.60E-05	Epithelial

hsa-miR-7	64	8	8	5.00E-06	Epithelial

hsa-miR-500	16	2	8	5.10E-07	Epithelial

hsa-miR-210	128	16	8	1.30E-05	Epithelial

hsa-miR-892b	16	2	8	4.10E-09	Epithelial

hsa-miR-200b*	32	2	16	1.50E-08	Epithelial

hsa-miR-196a	64	2	32	5.90E-07	Epithelial

hsa-miR-192	2048	128	16	1.60E-07	Epithelial

hsa-miR-192*	64	2	32	1.40E-10	Epithelial

hsa-miR-96	64	2	32	9.60E-06	Epithelial

hsa-miR-203	128	2	64	2.70E-08	Epithelial

hsa-miR-215	1024	32	32	1.20E-06	Epithelial

hsa-miR-375	128	4	32	1.00E-05	Epithelial

hsa-miR-194	2048	16	128	1.20E-06	Epithelial

hsa-miR-429	512	4	128	3.80E-08	Epithelial

hsa-miR-200b	2048	32	64	1.60E-05	Epithelial

hsa-miR-141	1024	8	128	6.60E-07	Epithelial

hsa-miR-200a	1024	8	128	7.90E-07	Epithelial

hsa-miR-200c	1024	8	128	1.10E-05	Epithelial

We then assessed the miRNA expression profiles of the colorectal tumors in the epithelial cells and identified 26 miRNAs that could differentiate adenomas from normal and carcinoma tissues (Figure 4B, Additional file 3). We further evaluated the miRNA profiles of the colorectal tumors in the stromal cells and identified 21 differentially expressed miRNAs that separated normal-adenomas into one group and carcinomas into another group (Figure 4C, Additional file 4). We finally examined the miRNA profiles of the colorectal tumors in the mixed cell types (epithelial and stromal cells) and identified 46 differentially expressed miRNAs amongst normal, adenoma and carcinoma tissues (Figure 4D, Additional file 5). The similar cases were observed in both stromal and mixed cell types where the miRNAs could not separate adenomas from normal tissues.

We compared the expression profiles of 5 miRNAs in colorectal tumors with data previously published [24]. Schetter *et al*. used whole colorectal tissues while our study used LCM selected epithelial cells. Using the whole colorectal tissues, significant fold changes were identified in only one miRNA for adenomas and 5 miRNAs for carcinomas, while considerable changes were seen in 3 miRNAs for adenomas and 4 miRNAs for carcinomas when we used the pure epithelial cells (Table 3). The overall fold-changes obtained on the whole colorectal tissues were considerably lower than those determined using the pure epithelial cells.

**Table 3:** Comparison of miRNA expression profiles between mixed and epithelial cell types of colorectal tumors

Name	Mixed cell types^a^		Epithelial cell type^c^	
	p value^b^	fold change	p value^d^	fold change
**Adenoma vs. paired nontumorous tissue**				

hsa-miR-20a	0.82	0.9	0.021	1.5

hsa-miR-21	0.006	1.6	0.004	2.2

hsa-miR-106a	0.19	1.2	0.658	1.3

hsa-miR-181b	0.27	1.2	0.702	0.8

hsa-miR-203	0.14	1.7	0.001	3.2

**Carcinoma vs. paired nontumorous tissue**				

hsa-miR-20a	< 0.001	2.3	0.02	2.9

hsa-miR-21	< 0.001	2.8	0.003	2.3

hsa-miR-106a	< 0.001	2.4	0.032	4.6

hsa-miR-181b	< 0.001	1.4	0.044	2.1

hsa-miR-203	< 0.001	1.8	0.248	1.8

^a ^the data obtained from the previous report by Schetter *et al*. [24].

^b ^Wilcoxon matched pairs test.

^c ^the data obtained from this study.

^d ^Matched pairs test.

### Across-platform comparison

**Figure 5:**
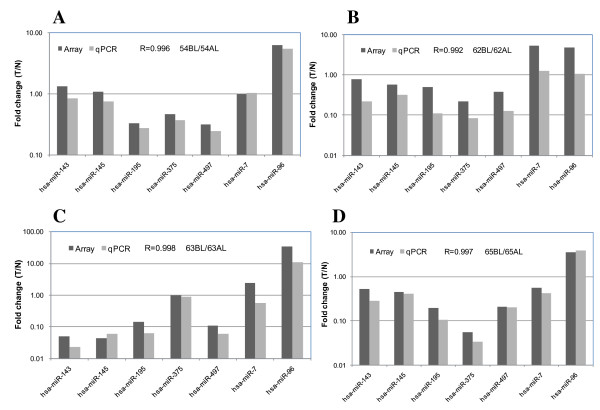
**Across-platform comparison**. A) comparison of the fold changes in sample pair 54 determined by Agilent miRNA microarrays and by quantitative RT-PCR (54AL: LCM-selected epithelial cells of normal colorectal tissue; 54BL: LCM-selected epithelial cells of Dukes' B carcinomas); B) comparison of the fold changes in sample pair 62 determined by Agilent miRNA microarrays and by quantitative RT-PCR (62AL: LCM-selected epithelial cells of normal colorectal tissue; 62BL: LCM-selected epithelial cells of Dukes' B carcinoma); C) comparison of the fold changes in sample pair 63 determined by Agilent miRNA microarrays and by quantitative RT-PCR (63AL: LCM-selected epithelial cells of normal colorectal tissue; 63BL: LCM-selected epithelial cells of Dukes' C carcinoma) and D) comparison of the fold changes in the sample pair 65 determined by Agilent miRNA microarrays and by quantitative RT-PCR (65AL: LCM-selected epithelial cells of normal colorectal tissue; 65BL: LCM-selected epithelial cells of Dukes' D carcinoma). R indicates the average correlation of 7 individual miRNAs.

To examine consistency with other platform, data from quantitative RT-PCR were generated on 7 miRNAs using 8 LCM-selected epithelial cells derived from 4 pairs of colorectal tumor tissues. The correlation (R) of fold changes between Agilent miRNA microarrays and quantitative RT-PCR was 0.996. The expression patterns of the miRNAs for 4 pairs of the colorectal tumors are shown in Figure 5. The results demonstrate that the miRNA signatures discovered using Agilent miRNA microarrays are highly reliable.

## Discussion

Combination of LCM with genome-wide miRNA analysis has not been widely applied to discover miRNA biomarkers in solid tumors. This is due to the facts that the tiny amounts of miRNA present in the cells (~0.001-0.1% of total RNA) and RNA recovered from LCM is typically very poor in both quality and quantity using conventional LCM procedures [25]. RNA degradation is primarily due to endogenous RNases that are activated in an aqueous environment. Based on this observation, we tested ethanol fixation and RNase inhibitor treatment on tissue preparations to procure high-quality yields of RNA. We used ethanol fixation to minimize the tissue sections for exposure to water, whereas RNase inhibitor treatment was used to inhibit the reactivation of endogenous RNases during the staining process.

We found that ethanol fixation of tissue sections is the preferred procedure for ensuring the highest quality and yield of RNA from LCM. To maximize the balance between tissue morphology and RNA quality, the sections on the slides should be immediately fixed with 100% ethanol at -25°C in the cryostat for 10 minutes, and then stored at -80°C for 2 hours. This ethanol fixation procedure produced a 1.8-fold improvement in RNA quality and a 1.5-fold increase in RNA quantity compared to the sections without the fixation. In agreement with previous reports discussing LCM protocols [26], the sections should not be dried on the slide at room temperature. All reagents for fixation, staining and dehydration should be cooled to 4°C. The staining should be carried out on ice.

The efficacy of RNase inhibitor treatment on tissue sections during the staining process is uncertain. Kube *et al*. reported that RNase inhibitors could significantly improve RNA quality [27], however, we did not observe considerable improvement on either RNA quality or quantity in the sections treated with RNase inhibitor. In agreement with our observation, a recent study revealed no difference in the quantity and quality of RNA recovered from microdissected colon cancer samples with and without RNase inhibitor treatment [28].

It has been shown that RNA can be damaged by heat, UV light, chemical components of histological staining and enzymatic degradation. Therefore, LCM itself can affect the quality of total RNA. On the other hand, the procedure of tissue dissection will destroy the integrity of cells especially in an aqueous environment, meanwhile, endogenous RNases will be released and have much chances to connect with RNA. In our study, applying LCM lowered 30% of the RIN values demonstrates that LCM can introduce RNA damage during its procedure. It is crucial to thoroughly air-dry the slides before placing them into the LCM instrument for preserving RNA degradation.

We could not directly compare RNA quantity in the tissue sections with and without LCM. Using our improved protocol for tissue preparation, the yield of total RNA from ~2 × 10^5 ^LCM selected epithelial cells was between 500 and 1500 ng. The time required to select and capture such an amount of relevant cells is usually 1-2 hours when isolating cells located in a complex tissue. The order of RNA quality after LCM is carcinoma (7.2 ± 1.2) > adenoma (5.7 ± 1.1) > normal tissue (5.2 ± 1.5). This could be due to the fact that RNase activity in the carcinoma tissue is lower than that in the normal tissue [29].

Recently, Ibberson et al. reported that RNA degradation compromised the reliability of miRNA expression profiling and stated that total RNA degradation with RIN values less than 7 should not be used for analysis of individual miRNAs [30]. In our study, we used a mirVana miRNA Isolation kit to prepare total RNA for the miRNA analysis. The RNA isolation procedure combining the advantages of organic extraction and solid-phase extraction can effectively recover small RNAs. The RNA quality recovered from our LCM and isolation procedures is 37% with RIN ≥ 7, 39% with RIN ≥ 5 and 24% with RIN < 5. To minimize experimental variations, we chose to use Agilent miRNA microarray, since the platform features the direct end-labeling and profiling of mature miRNAs from total RNA without any size fractionation or amplification. Obviously, the different RNA isolation methods and microarray platforms used in the studies can affect the RNA quality and thus the miRNA profiles. The tissues shall be procured immediately after surgery, cut into small pieces (~1 cm^2 ^× 0.5 cm), embedded in OCT compound, fast-frozen in liquid nitrogen and stored at -80°C. Using such frozen tissue processing, the RNA quality of our frozen tissues is 79% with RIN ≥ 7 and 21% with RIN ≥ 5. Methods upstream of RNA isolation are crucial for preserving RNA integrity.

We determined the correlation of LCM selected epithelial cells derived from the same tissue type and variability of the triplicate LCM experiments using the microarray platform. We show that the correlation of the individual samples from the same tissue type is between 0.937 and 0.963, while the correlation amongst the triplicate LCM experiments is 0.999. We further applied quantitative RT-PCR to cross-verify the expression patterns of 7 different miRNAs using 8 LCM selected epithelial cells. The correlation of the fold changes between Agilent miRNA microarray and quantitative RT-PCR are excellent (R = 0.996). The highly reproducible data demonstrate that RNA quality with RIN value ≥ 5 obtained from our LCM and RNA isolation procedures is generally sufficient for genome-wide miRNA analysis.

High-throughput microarrays have significantly enhanced our knowledge of cancer biology [31-33]. The accuracy of microarray data, however, is determined by the specificity of the input RNA. We can imagine difficulties arising during microarray analysis of tissue when there are varying levels of tumor cells versus normal and stromal cells. For example, a tissue comprised of 60% tumor, 30% normal and 10% stromal cells, would have non-cancer cell types contributing to more than 40% of the overall signal. Since the amount of non-cancer tissue in colorectal tumors is highly variable (20-80%), taking a "whole tissue" approach to microarrays may yield great variations in the results, as compared to those attainable using LCM selection of only epithelial cancer cells. In our study on 18 colorectal tissues, we found that the miRNAs in the epithelial cells could differentiate three categories of colorectal tissues (normal, adenoma and carcinoma), however, the miRNAs in both stromal and mixed cell types could not separate adenomas from normal tissues. When we compared the expression profiles of 5 miRNAs from the report of Schetter *et al*. [24] with those collected in our study, we found that the overall fold-changes obtained on the whole tissues were considerably lower than those determined by using the pure epithelial cells, especially in the case of adenomas. For carcinomas, we observed the significant concordance of the regulation trends between the two studies, although the total RNA source and microarray platforms used for both experiments were not identical. For adenomas, we found the considerable differences of the fold changes between the whole colorectal tissues and LCM-selected epithelial cells. Such differences observed in both studies are mainly due to the varying levels of the tumor cells in the whole tumor tissues. In some cases, the tumor cells may be less than 10%. It is clear that the expression levels of the whole tumor tissue cannot only represent the signals from the tumor cells, but also from the normal epithelial cells and other cell types of interstitial tissue. This demonstrates the potential power of discovering miRNA biomarkers in a complex tissue using the combination of LCM with genome-wide miRNA analysis.

Most cancers are epithelial in origin and arise through a stepwise progression from normal cells, through dysplasic cells, into malignant cells [34]. Focusing research on cell-specific molecular biomarkers can help in the development of novel concepts for diagnosis and treatment of epithelial cancers. In our study, we discovered 51 differentially expressed miRNAs in the pure epithelial and stromal cells. The miRNAs that are specifically expressed in the epithelial cells hold potential utility in the further discovery of cell-specific miRNA biomarkers for epithelial cancers. Other miRNAs that are highly expressed in the stromal cells might have values in the establishment of their roles on immune function and relate to cancer progression and recurrence in solid tumors [35]. Our small study presents a good example using the microdissection for elucidation of miRNA biomarkers in specific cell populations. Additional confirmatory studies, however, are required to establish the full significance of our findings.

## Conclusions

Our results show good quality and quantity of RNA recovered from LCM using our improved procedures on tissue preparation. By comparing the miRNA expression profiles of colorectal tissues in the mixed cell types with the pure epithelial cells, we demonstrate the feasibility and potential power of discovering miRNA biomarkers in complex tissue using the combination of LCM with genome-wide miRNA analysis. Additionally, we discovered 51 differentially expressed miRNAs in epithelial and stromal cells. Such cell type-specific miRNAs have great potentials in the development of novel approaches for diagnosis and treatment of epithelial cancers. We expect that our optimized ethanol-fixation protocol will serve as a basic tool for molecular analysis of frozen tissues. The protocol is simple and shall be easy to implement in a standard biology laboratory.

## Methods

### Frozen tissue sections

Colorectal tissues were obtained from the tissue bank at Shanghai Medical College in Fudan University. All patients who participated in the study had given informed consent. The collection of the tissue specimens in accordance with the protocol was approved by the Institutional Review Board of Shanghai Medical College. The tissues were procured immediately after surgery, cut into the size of ~1 cm^2 ^× 0.5 cm pieces, embedded in optimum cutting temperature (OCT) compound, fast-frozen in liquid nitrogen and stored at -80°C. Serial cryostat sections (10 μm) were cut at -25°C by using SLEE Cryostat MNT Instrument (SLEE Medical GMBH, Mainz, Germany) and placed onto membrane slides (Arcturus Veritas, Mountain View, CA).

### Tissue preparation and LCM

Six different experiments on tissue preparations were performed to determine the effects of tissue manipulations on RNA quality and quantity (Table 1). Each experiment was performed in duplicate for each of three colorectal tissues. In total, six replicates were performed per experiment (two technical and three biological replicates). Concentration of RNase Inhibitor used in the hemotoxylin solution was according to the manufacturer's specified instruction (Promega, Madison, WI).

For the improved procedures on tissue preparation, the sections were immediately fixed with 100% ethanol in the cryostat at -25°C for 10 minutes and the slides were stored in 100% ethanol at -80°C for an additional 2 hours. The slides were then washed with DEPC treated water on ice for 30 seconds and stained with MHS128 hematoxylin solution (Sigma-Aldrich, St. Louis, MO) for 1 minute. Finally, the slides were dehydrated with 100% ethanol for 30 seconds and xylene for 5 minutes and subsequently air-dried.

**Figure 6:**
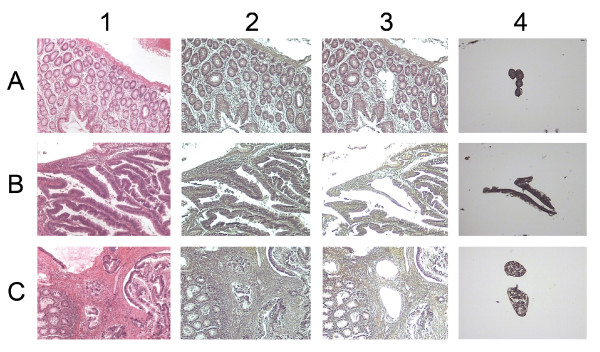
**Laser capture microdissection of colorectal cells**. A) normal; B) adenoma and C) carcinoma. 1) H&E-stained slide (× 20); 2) hematoxylin stained slide before LCM (× 20); 3) hematoxylin stained slide after LCM (× 20) and 4) cap showing adherent cells (× 20).

The stained slides were placed into a Veritas Microdissection Instrument (Arcturus Veritas, Mountain View, CA). The cells of interest (~2 × 10^5^) were selected and captured using ultraviolet laser cutting following the manufacturer's recommended protocol. The LCM cap was immediately placed in a microcentrifuge tube containing 400 μL Lysis/Binding Buffer (Ambion, Austin, TX), which was vortex mixed and stored upside down at -80°C until RNA isolation. The essential protocol and reagents for LCM were from Arcturus (Mountain View, CA). For each tissue, hematoxylin and eosin (H&E)-stained frozen sections were prepared to guide the area of interest for LCM. Examples of LCM isolated cells from colorectal tissues are shown in Figure 6 with a schematic depiction of the improved LCM protocol shown in Additional file 6.

Using the improved protocol for tissue preparation, LCM was performed on 11 tissue sections (n = 4 normal, n = 3 adenomas and n = 4 carcinomas) to compare the RNA quality with and without LCM (Table 1). Additionally, LCM was performed on 101 colorectal tissues (43 normal, 14 adenomas and 44 carcinomas) to examine the consistency of RNA quality recovered from LCM. Furthermore, LCM was performed on 18 colorectal tissues (n = 6 normal, n = 6 adenomas and n = 6 carcinomas) to isolate epithelial and stromal cells for genome-wide miRNA analysis. Finally, triplicate LCM experiments (~1 × 10^5 ^per LCM) were independently performed on one Dukes' B carcinoma to isolate epithelial cells for determining the variability of the LCM protocol.

### RNA isolation and quality control

Total RNA was extracted from the tissue sections by using mirVana miRNA isolation kit according to the instructions from the manufacturer (Applied Biosystems, Foster City, CA). The concentration was quantified by NanoDrop 1000 Spectrophotometer (NanoDrop Technologies, Waltham, MA). The quality control of RNA was performed by a 2100 Bioanalyzer using the RNA 6000 Pico LabChip kit (Agilent Technologies, Santa Clara, CA). The quality was measured by using RNA integrity number (RIN). A RIN score was generated for each sample on a scale of 1-10 as an indication of RNA quality [36,37]. The quality is considered to be excellent for RIN >/= 7-10, good for RIN >/= 5 and poor for RIN < 5. Variation of RNA quality and associated RIN scores are displayed in Additional file 7.

### Genome-wide miRNA analysis

Human miRNA microarrays (Agilent Technologies, Santa Clara, CA) were used to compare the expression profiles of 18 colorectal tissues (n = 6 normal, n = 6 adenomas and n = 6 carcinomas) between LCM selected epithelial cells versus stromal cells. Furthermore, the microarray platform was used to determine the reproducibility of the LCM protocol using the pure epithelial cells isolated from 61 colorectal tissues (n = 24 normal, n = 13 tubular adenomas and n = 24 Dukes' C carcinomas). The microarray contains probes for 723 human miRNAs from the Sanger database v.10.1. Total RNA (100 ng) derived from each of the colorectal samples were used as inputs for labeling via Cy3 incorporation. Microarray slides were scanned by XDR Scan (PMT100, PMT5). The labeling and hybridization were performed at Shanghai Biochip Company according to the protocols in the Agilent miRNA microarray system.

### Quantitative RT-PCR

Quantitative RT-PCR was performed using Taqman MicroRNA assays (Applied Biosystems, Foster City, CA) according to the manufacturer's instructions with the Light Cycling 480 system (Roche Applied Science, Indianapolis). The assays were performed for 7 miRNAs (hsa-miR-143, hsa-miR-145, hsa-miR-195, hsa-miR-375, hsa-miR-497, hsa-miR-7 and hsa-miR-96) using 8 LCM-selected epithelial cells derived from 4 pairs of colorectal tumor tissues. The expression level of the small nuclear RNA U47 was used as the normalization control. All assays were carried out in triplicate.

### Data analysis

#### Significance analysis of different tissue preparations

Paired t-test was performed on RNA quality and quantity data derived from the different tissue preparations. The standard deviation (SD) of the replicate experiments was determined to assess the variability of the tissue preparations.

#### Differential miRNA expression analysis

The microarray image information was converted into spot intensity values using Scanner Control Software Rev. 7.0 (Agilent Technologies, Santa Clara, CA). The signal after background subtraction was exported directly into the GeneSpring GX10 software (Agilent Technologies, Santa Clara, CA) for quantile normalization. The mean normalized signal from biological replicates was used for comparative expression analysis. Unpaired t-test with Benjamini-Hochberg correction (p value </= 0.001) was used to identify differentially expressed miRNAs between epithelial and stromal cells. One-way analysis of variance (ANOVA) with a p value </= 0.05 was performed to determine differentially expressed miRNAs amongst normal, adenoma and carcinoma tissues. Hierarchical clustering was performed with Pearson correlation using the differentially expressed miRNAs. The fold changes of expression signals between normal and tumor samples were calculated from the normalized values.

### Correlation analysis

Pearson correlation was performed with all of 723 human miRNAs after the quantile normalization. The correlation (R) between individual samples in the same tissue type was determined using the normalized signals.

## Authors' contributions

The study was designed and organised by HZ and YW. The experimental work was carried out by SW, LW, TZ and XG with coordination by HZ and YW. The statistical analysis was performed by JL. The manuscript was initially drafted by SW. YW organized the final versions of the manuscript and arranged submission. All authors read and approved the final manuscript.

## Supplementary Material

Additional file 1 **Effect of RNase inhibitor on RNA quality and quantity**. A) RNA quality (RIN scores) of tissue sections in the presence (n = 6) and absence (n = 6) of RNase inhibitors and B) RNA quantity (ng) of the tissue sections in the presence (n = 6) and absence (n = 6) of RNase inhibitor. S1, S5 and S6 indicates sample 1, 5 and 6 respectively. Error bars represent the corresponding SD. Click here for file

Additional file 2 **Expression levels of 723 human miRNAs in LCM selected epithelial and stromal cells from colorectal tissue**. The table lists the mean normalized signals, the corresponding SD and fold changes of 723 human miRNAs in LCM selected epithelial and stromal cells of colorectal normal (n = 6), adenoma (n = 6) and carcinoma tissues (n = 6). Click here for file

Additional file 3 **Differentially expressed miRNAs in LCM selected epithelial cells from colorectal tissue**. The table lists the mean normalized signals, fold changes and ANOVA p-values of 26 differentially expressed miRNAs in LCM selected epithelial cells of colorectal normal (n = 6), adenoma (n = 6) and carcinoma tissues (n = 6). Click here for file

Additional file 4 **Differentially expressed miRNAs in LCM selected stromal cells from colorectal tissue**. The table lists the mean normalized signals, fold changes and ANOVA p-values of 21 differentially expressed miRNAs in LCM selected stromal cells of colorectal normal (n = 6), adenoma (n = 6) and carcinoma tissues (n = 6). Click here for file

Additional file 5 **Differentially expressed miRNAs in the mixed cell types (epithelial and stromal cells) from colorectal tissue**. The table lists the mean normalized signals, fold changes and ANOVA p-values of 46 differentially expressed miRNAs in the mixed cell types (epithelial and stromal cells) of colorectal normal (n = 6), adenoma (n = 6) and carcinoma tissues (n = 6). Click here for file

Additional file 6 **Schematic depiction of the improved protocol on tissue preparation for laser capture microdissection**. The figure shows the procedures of the optimized ethanol-fixation protocol on tissue preparation for LCM. Click here for file

Additional file 7 **Variation of RNA quality and its associated RIN score**. A) gel electrophoresis patterns of total RNA samples with various RNA quality and B) electropherograms of total RNA samples with associated RIN scores. Click here for file
