# Supplementary material for: Improvement of tissue preparation for laser capture microdissection: application for cell type-specific miRNA expression profiling in colorectal tumors
Source: BMC Genomics. 2010 Mar 10;11:163. doi: 10.1186/1471-2164-11-163 (PMC2853520; doi:10.1186/1471-2164-11-163)
Supplement: Additional file 1 — Effect of RNase inhibitor on RNA quality and quantity. A) RNA quality (RIN scores) of tissue sections in the presence (n = 6) and absence (n = 6) of RNase inhibitors and B) RNA quantity (ng) of the tissue sections in the presence (n = 6) and absence (n = 6) of RNase inhibitor. S1, S5 and S6 indicates sample 1, 5 and 6 respectively. Error bars represent the corresponding SD. [file 1471-2164-11-163-S1.DOC]

**Additional file 1**

***Effect of RNase inhibitor on RNA quality and quantity****. A) RNA quality (RIN scores) of tissue sections in the presence (n=6) and absence (n=6) of RNase inhibitors and B) RNA quantity (ng) of the tissue sections in the presence (n=6) and absence (n=6) of RNase inhibitor. S1, S5 and S6 indicates sample 1, 5 and 6 respectively. Error bars represent the corresponding SD.*

*
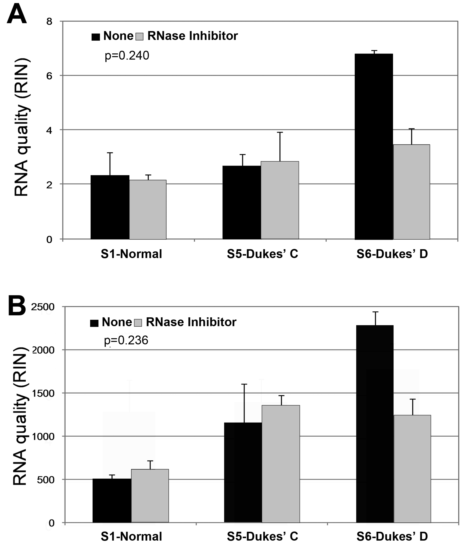
*
