# Supplementary material for: Improvement of tissue preparation for laser capture microdissection: application for cell type-specific miRNA expression profiling in colorectal tumors
Source: BMC Genomics. 2010 Mar 10;11:163. doi: 10.1186/1471-2164-11-163 (PMC2853520; doi:10.1186/1471-2164-11-163)
Supplement: Additional file 6 — Schematic depiction of the improved protocol on tissue preparation for laser capture microdissection. The figure shows the procedures of the optimized ethanol-fixation protocol on tissue preparation for LCM. [file 1471-2164-11-163-S6.DOC]

**Additional file 6**

***Schematic depiction of the improved protocol on tissue preparation for laser capture microdissection.*** *The figure shows the procedures of the optimized ethanol-fixation protocol on tissue preparation for LCM.*

Cut 10-μm frozen tissue sections and place onto membrane slide

Fix the slide immediately in 100% ethanol for 10 min and store at -80℃ for 2 h

Wash the slide with DEPC treated water for 30 s

Stain the slide with hematoxylin for 1 min

Dehydrate the slide with 100% ethanol for 30 s and xylene for 5 min

Air-dry the slide and place the slide into LCM instrument

Select interested cells ~ 2x10^5^

Capture the selected cells using UV- LCM
