# Supplementary material for: Improvement of tissue preparation for laser capture microdissection: application for cell type-specific miRNA expression profiling in colorectal tumors
Source: BMC Genomics. 2010 Mar 10;11:163. doi: 10.1186/1471-2164-11-163 (PMC2853520; doi:10.1186/1471-2164-11-163)
Supplement: Additional file 7 — Variation of RNA quality and its associated RIN score. A) gel electrophoresis patterns of total RNA samples with various RNA quality and B) electropherograms of total RNA samples with associated RIN scores. [file 1471-2164-11-163-S7.DOC]

### Additional file 7

### *Variation of RNA quality and its associated RIN score. A) gel electrophoresis patterns of total RNA samples with various RNA quality and B) electropherograms of total RNA samples with associated RIN scores.*

**A**

**B**

Rin 5.2 7.1 7.5

**
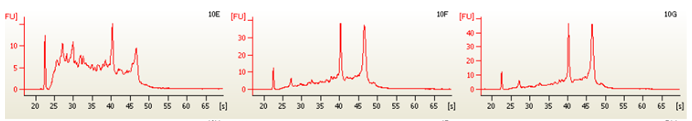
**

Rin 7.4 2.5 6.1

**
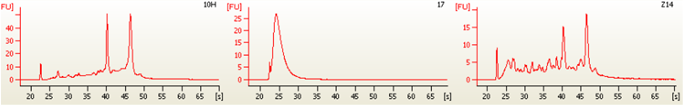
**
